# Overview of the Influence of Silver, Gold, and Titanium Nanoparticles on the Physical Properties of PEDOT:PSS-Coated Cotton Fabrics

**DOI:** 10.3390/nano12091609

**Published:** 2022-05-09

**Authors:** Fahad Alhashmi Alamer, Rawan F. Beyari

**Affiliations:** Department of Physics, Faculty of Applied Science, Umm AL-Qura University, Al Taif Road, Makkah 24382, Saudi Arabia; beyari.f.rawan23@hotmail.com

**Keywords:** metallic nanoparticles, titanium nanoparticles, PEDOT:PSS, conductive polymers, e-textile

## Abstract

Metallic nanoparticles have been of interest to scientists, and they are now widely used in biomedical and engineering applications. The importance, categorization, and characterization of silver nanoparticles, gold nanoparticles, and titanium nanoparticles have been discussed. Poly(3,4-ethylenedioxythiophene) poly(styrenesulfonate) (PEDOT:PSS) is the most practical and reliable conductive polymer used in the manufacturing of conductive textiles. The effects of metallic nanoparticles on the performance of PEDOT:PSS thin films are discussed. The results indicated that the properties of PEDOT:PSS significantly depended on the synthesis technique, doping, post-treatment, and composite material. Further, electronic textiles known as smart textiles have recently gained popularity, and they offer a wide range of applications. This review provides an overview of the effects of nanoparticles on the physical properties of PEDOT:PSS-coated cotton fabrics.

## 1. Introduction

### 1.1. Nanoparticles

**Figure 1:**
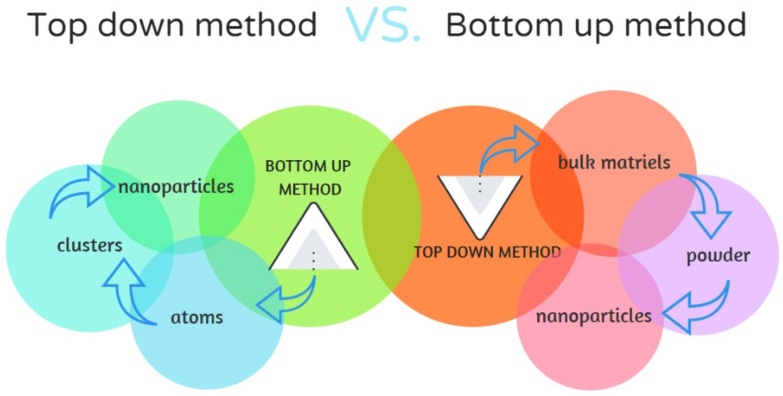
The difference between the bottom-up method and top-down method.

Nanotechnology is a new branch of science that deals with the synthesis and development of various nanomaterials containing nanoparticles (NPs) with a size of 1–100 nm, which, in at least one dimension, have a length of 1–1000 nm and a diameter of 1–100 nm [1]. In recent years, the use of NPs has increased considerably in various fields, including electronics, environmental science, human reproduction, pharmaceuticals, and medicine. Further, they have been extensively utilized in the mechanical industry, energy production [2], and in the production of materials with novel properties. Different metallic nanostructures are synthesized using metals such as copper, zinc, titanium, magnesium, gold, and silver. The production of metallic NPs is based on the bottom-up or top-down methods and depends on the starting material as shown in Figure 1 [3].

The primary difference between the two methods is the starting material used for NP fabrication. Bottom-up fabrication requires the assembly of atoms or molecular components as opposed to the top-down approach, wherein small patterns are etched from the bulk material [4]. The synthesis of metal NPs, nanostructures, and nanomaterials has gained research interest because of their striking properties that are particularly useful for catalysis. The NPs and nanostructured materials are an active research area and a techno-economic sector completely developed in several applications. They gained prominence because of their excellent mechanical and physicochemical properties such as a low melting point, wettability, electrical and thermal conductivity, catalytic activity, light absorption, and scattering, and this results in a better performance than their solid counterparts [5].

### 1.2. Importance of Metallic NPs

Metallic NPs are a highly researched and diverse class of materials with a wide range of applications. The presence of NPs in the environment has grave consequences on human health and ecosystems. The size, size distribution, shape, structure, microstructure, content, and homogeneity of the particles are key factors in assessing the potential impact of such materials on the human health and environment [6]. Thus, several approaches for identifying anthropogenic and naturally occurring NPs have been developed. Understanding the context in which NPs are discovered or used is critical for elucidating their importance in the environment. NPs composed of ferrous metals are used to clean sites polluted with chlorinated hydrocarbons and other anthropogenic pollutants [7,8]. Metal-enriched NPs have attracted considerable interest in industry and medicine. NPs based on noble metals such as gold, silver, platinum, and palladium are becoming increasingly popular because of their unusual electrical, optical, and electronic capabilities, and their catalytic properties [9]. Physical and chemical approaches can be used to fabricate metal NPs with varying sizes. For example, metal NPs capped with thiols can be converted into ordered one-, two-, and three-dimensional structures, which can be used in nanodevices [10].

Dara et al. [11] combined materials science and biology to develop a green synthesis method for metal NPs for a variety of applications. However, the synthesis of metallic NPs remains problematic under several circumstances [12]. As the most common method for the preparation of metallic NPs [13], vacuum evaporation uses a low-pressure thermal effect and is effective only for elements with a low melting point and high saturation vapor pressure [14]. Metal and oxide NPs are commonly used as antibacterial ingredients in textiles; they can prevent the growth of bacteria and germs and even kill them, which helps reduce the spread of infectious diseases through chemical or physical treatments [15]. Ag, Au, Cu, Ti, and Zn NPs play an important role in nanomaterial research and technology because of their remarkable and unique optical, mechanical, and catalytic properties; conductivity; heat transfer; low toxicity; high stability; biocompatibility; and bactericidal activity [16]. 

### 1.3. Titanium Nanoparticles

Titanium nanoparticles (Ti NPs) are widely used nanoscale metals because of their mechanical strength, biocompatibility, and long history of use of titanium [17]. Titanium is employed as an inert bio-implant material in the medical field, and its alloys have been used to create a variety of implantation and fixation devices. Further, it has several industrial applications such as pharmaceutical coatings, gum and confections processing materials, food additives, and paints. However, concerns regarding the negative effects of titanium buildup and its impact on the human body have arisen because of its extensive use [18,19]. The number of studies on the toxicity of titanium, especially in the medical field, has increased in recent years; however, these studies have primarily focused on environmental and fundamental issues. Consequently, there is a need to increase awareness regarding the safety and risks of titanium in the medical field [20].

Alzubi et al. [21] shed light on the influence of the size of NPs on their structural properties. They synthesized Ti NPs using a physical vapor deposition method with an electron beam. Ti NPs could be generated with varying diameters by adjusting the thickness of the deposited layers to different values during the production process. The results indicated that the diameter of the Ti NPs also increased with an increase in the thickness of the formed layer. The thickness of the deposited layer affected the coverage and separation of NPs at different deposited thicknesses. Titanium is a particularly fascinating element because of its remarkable resistance to corrosion [22]. Further, Ti NPs formed at the metal−tissue contact may contribute to the long-term durability of titanium-based biomedical implants. Ghosh et al. [23] demonstrated that the structure of nanocrystals could be clearly revealed using high-resolution transmission electron microscopy and demonstrated the production of stable nanometer-sized titanium particles. Titanium dioxide (TiO_2_) is the most commonly used semiconductor in the form of NPs, nanowires, and nanofibers [24]. TiO_2_ NPs have a significant application potential because of their fascinating optical, electrical, and photocatalytic properties, and their affordability, safety, ability to filter contaminants, chemical stability, and non-toxicity. The addition of TiO_2_ NPs to textiles has resulted in several new and improved properties. Furthermore, TiO_2_ NPs have been used in the fabrication of textile flame retardants [25]. In addition, TiO_2_ has proven to be an attractive antibacterial agent. A previous study revealed that it exhibits potent antifungal and antibacterial activities against a variety of Gram-positive and Gram-negative bacteria in several tests [26]. Thus, TiO_2_ NPs with drastically improved properties have been developed.

### 1.4. Silver Nanoparticles

Noble metal NPs, especially silver nanoparticles (Ag NPs), have been used in various applications for several years. Their outstanding properties including biological functions facilitate their use in the biomedical field and in the catalysis [27]. Compared with solid silver, Ag NPs possess unique biological, chemical, and physical properties. The optical behavior, enhanced electrical conductivity, excellent thermal and chemical stability, nonlinearity, and catalytic activity are some of the chemical and physical properties of Ag NPs. The properties of Ag NPs have been exploited in electronics and medical applications. In the antibacterial field, they were widely used to treat microorganisms such as fungi, viruses, and bacteria. In the textile industry, they are applied in water filter membranes as a protective shield against bacteria and other pathogens present in water because of their delayed-release properties [28]. Nanomedicine, drug delivery, cosmetics, electrical applications, and environmental protection have all benefited from the potential use of Ag NPs. Silver is important in human health and disease treatment because of its potent antibacterial properties, which aid in wound healing, skincare, and water purification. Ag NPs rapidly interact with microbial membranes and kill cells to prevent or treat infectious diseases. In addition, the most common method for producing silver NPs is a chemical reduction with inorganic and organic reducing agents such as hydrazine [29]. 

Mehravani et al. [30] reported the preparation of silver and chitosan NPs, and their antibacterial and cytotoxic properties when applied to a tissue. Antibacterial and cytotoxic assays demonstrated that the Ag NPs exhibited a strong antibacterial effect on both Gram-positive and Gram-negative bacteria and exhibited no cytotoxicity; the antibacterial effect was noticeable even after ten washes. In addition, studies have revealed that green approaches to preparing NPs are more successful than physical and chemical syntheses because they carry fewer risks of failure are less expensive, and they are easier to characterize. The environment is exposed to a number of stressors because of the hazardous metabolites produced during the physical and chemical synthesis of NPs. Thus, this method has attracted attention over the last decade, especially for the preparation of Ag [31]. Green synthesis is a biocompatible and environmentally friendly process that uses a cap stabilizer and plant extracts, yeast, or bacteria to regulate the size and prevent agglomeration [32]. In recent years, Ag NPs with tunable physical and chemical properties have been extensively studied to improve their applicability because of their low toxicity and biocompatibility. Moreover, highly conductive Ag NPs have been used to create electrocardiographs in wearable and flexible sensors. The excessive use of Ag NPs is cytotoxic, and their uncontrolled release into the environment can harm aquatic and terrestrial biota [33]. Chemical reduction, photochemical approaches, electrochemical methods, microwave processing, ultrasonic processing, and gamma irradiation are common methods employed in the fabrication of Ag NPs using inorganic salts as metal precursors. Most of these technologies have been successful in producing particles of the desired size and shape; however, they have certain drawbacks such as time-consuming preparation steps and material products that may be harmful to the environment [34].

### 1.5. Gold Nanoparticles

Gold nanoparticles (Au NPs) have been used for decades and are one of the most promising areas of current nanoscience and nanotechnology research [35]. They differ from loose gold in that loose gold is a yellow solid that is inert in nature, whereas Au NPs are a wine-red solution with antioxidant activities. Interactions between the particles and the formation of Au NP networks are important factors that determine the properties of these NPs [36]. The use of a single active component from a plant extract in the preparation of Au NPs is a crucial biosynthetic approach for purifying Au NPs and exploring their medical applications [37]. In addition to spherical Au NPs, other shapes of NPs, which include sub-octahedral, octahedral, decahedral, icosahedral, multi-twisted, irregular, tetrahedral, nanotriangles, nano prisms, hexagonal platelets, and nanorods can be prepared using appropriate techniques [38,39]. They are highly stable and can be synthesized as nanospheres, nanorods, nanocubes, nano-branches, nano-bipyramids, nano blooms, nano shells, nanowires, and nanocages [40,41]. Further, they possess various therapeutic applications, which include drug delivery systems for diseases such as cancer, cardiovascular diseases, and diabetes mellitus, as well as applications for the degradation of dyes in the environment, biosensors, and bioremediation of pollutants in the environment, soil, and atmosphere [42]. Further, they have multidisciplinary applications, which include medicine, materials science, biology, chemistry, and physics because of their unique optical and electrical properties and economic value [43]. There has been no systematic evaluation of their medical uses because Au NPs have wide applications in medicine. Therefore, researchers have proposed methods for synthesizing common Au NPs and characterizing them based on their different and diverse properties. Hu et al. [44] focused on the established medical uses of Au NPs. 

## 2. Toxicity of Metallic NPs

Nanotoxicology analyzes the toxicity of various nanomaterials in a range of micro- and macrospecies [45]. Various model species such as bacterial and algal monocultures, Daphnia, and human cell lines, have been used in the determination of toxicity [46]. The dispersion of NPs in different media was used in essays to estimate their toxicity. It is important to consider that different settings can cause different toxicities [47]. There are several different types of NPs, each of which possesses a number of properties. Depending on their size, shape, and surface reactivity, they can cause toxicity in human tissues and cell cultures, with biological toxicity being the most common. Different types of NPs, their importance in clinical settings, and a detailed understanding of the biological toxicity of NPs have been critically discussed previously [48]. Nonmetallic NPs and an increase in the degree of oxidation result in low toxicity to cells, whereas electronegativity, solubility, and the formation of a metal cation increase the cytotoxicity. A short hypothetical mechanism has also been discussed to determine the reason for cell toxicity caused by MeOx NPs [49]. The toxicity of heavy metals caused by their hazardous nature, bioaccumulation, and biodegradability has emerged as a serious problem. Living things are exposed to these heavy metals from various sources; however, water, especially drinking water, is a major source of heavy metals. Several studies have been conducted on the removal of these heavy metals from water. However, conventional techniques possess a number of drawbacks, which includes inefficiency and environmental impact. Magnetic iron oxide NPs have emerged as the adsorbent of choice for water treatment. They offer several advantages, which include environmental friendliness, cost efficiency, ease of application, regeneration, and surface modification [50]. Thorough toxicity studies and comparisons of the advantages and disadvantages of cobalt ferrite NPs as surface modifiers are limited in the literature and should be considered in future research. Mmelesi et al. [51] provided information on the status of cobalt ferrite NPs and nanocomposites in terms of wastewater treatment applications, their advantages, and toxicity. Toxicity is a critical consideration influenced by the shape, size, surface charge, and NP dosage when considering the potential uses of *Fusarium*-mediated NPs. *Fusarium* can be recommended as a promising and innovative fungus for NP production and potential biomedical and agricultural applications. Hou et al. [52] summarized the toxicity of TiO_2_ NPs on various organisms from different taxa such as microorganisms, algae, plants, invertebrates, and vertebrates. The results obtained provide new insights into the toxicity and mode of action of TiO_2_ NPs in living organisms that are useful for the risk assessment and safe use of TiO_2_ NPs.

## 3. Conductive Polymers

Conductive polymers (CPs) are popular organic polymers because of their unique electrical and optical properties. They possess material properties similar to those of certain metals and inorganic semiconductors, whereas retaining polymer properties, such as flexibility and ease of processing and synthesis, are commonly associated with traditional polymers [53]. CPs possess several advantages such as a light weight, processability, relatively high conductivity, stability, and flexibility, which make them well suited for the production of conductive textiles [54]. Since the discovery of metallic polymers [55] as bio transducers, intrinsically conductive polymers, and CP nanocomposites have been used. The advantages of CP composites include ease of molding, low density, a wide range of electrical conductivities, and corrosion resistance compared with the corresponding properties of metallic conductors. Metal wires can be directly blended with textile fibers or a coating of conductive materials, such as CPs, can be applied to create conductive fabrics [56]. CP composites are characterized by their electrical resistivity. For example, the CP composites often require an electrical resistivity of ~10^6^ Ω cm for electrostatic dissipation in plastic fuel tanks, whereas electromagnetic interference shielding requires an electrical resistivity of less than 10^−2^ Ω cm [57]. Threads coated with CPs obtained from commercially available yarns such as cotton, nylon, polyester, and silk are difficult to reduce because of their low chemical resistance and heat stability, which limits their use in several applications [58]. The ability of polymers to act as electrical insulators underpins their extensive use in electrical and electronic applications, wherein their resistance is approximately 10^15^ Wm [59]. In contrast, material developers have attempted to render polymers conductive by combining insulating polymers with conductive elements such as carbon black, carbon fibers, metal particles, or CPs such as polyaniline [60].

Numerous methods have been used to characterize CPs. For example, ultraviolet absorption spectroscopy is a highly sensitive method used for visualizing the electronic structure and nature of protonation in CPs and for distinguishing between conductive and nonconductive polymers [61]. Two- and four-probe techniques can be used to test the electrical resistivity of conductive fibers and small-diameter wires. The four-probe method is used to reduce the contact resistance when the fibers are highly conductive [62]. Furthermore, polymers have been used to improve the electrical conductivity of materials [63]. The thermal sintering of silver NPs with silver flakes dispersed in a polymer matrix at 180 °C was found to result in highly conductive polymer nanocomposites with a very low resistivity of 4.8 × 10^−5^ Ω cm, which suggests that the temperature at which the surface residues on the Ag NPs decompose has a significant effect on the sintering of Ag NPs. Zhang et al. [64] noted that electrical studies on the polymer nanocomposites have revealed that morphological changes produced by the sintering of Ag NPs with Ag flakes contribute significantly to lowering the contact resistance between the conductive fillers; this increases the conductivity of the nanocomposite.

### PEDOT:PSS Nano Composite Thin Films

**Figure 2:**
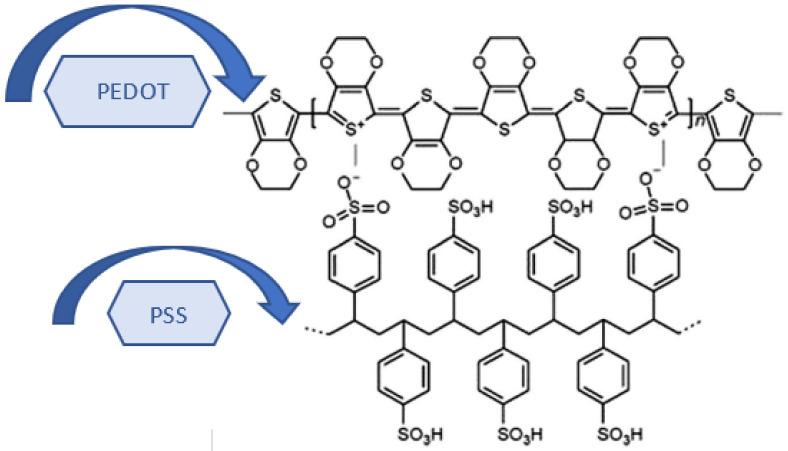
PEDOT:PSS chemical structure.

PEDOT:PSS (Figure 2) is an intrinsically conductive polymer and one of the most promising and successful CPs. Its advantages include environmental stability, mechanical flexibility, low cost, tunable electrical conductivity, and processability in solutions [65]. Blending an insulating composite material with a conductive component such as PEDOT:PSS is the most popular method for producing conductive fibers [66]. PEDOT:PSS exhibits excellent film-forming properties, high transparency in the visible range, and excellent thermal stability. In addition, the electrical conductivity of PEDOT:PSS films can be selectively tuned and improved by 2 to 3 orders of magnitude by doping with polar solvents and strong acids [67]. The best value of electrical conductivity reported in the literature is 4000 S cm^−1^. The doped PEDOT:PSS films are flexible and inherently stretchable to withstand mechanical testing. The PEDOT:PSS films have a work function of 4.8–5.4 eV, which makes them ideal for charge transfer and injection into optoelectronic devices as a p-type contact layer. Furthermore, it has attracted considerable attention because of the unique advantages of its conductive state such as low bandgap, superior electrochemical and thermal stability, and high transparency, which offer various useful applications for electronics, for organic or plastic electronic and optical devices [68,69]. However, PEDOT:PSS films in coating applications suffered from several drawbacks such as degradation and delamination in humid environments, which makes PEDOT:PSS unsuitable for applications in aqueous environments. Although stability was improved using ionic liquids, poly(ethylene glycol), and methylene ether, long-term stability was not reported in these studies. Wang et al. prepared the PEDOT:PSS composite films using poly(vinyl alcohol) and multi-walled carbon nanotubes. They found that films could be stabilized for several days; however, the composite had low electrical conductivity and poor optical transparency. Therefore, it remains a technical difficulty to produce a moisture-stable PEDOT:PSS film that simultaneously exhibits good electrical conductivity and optical clarity.

The PEDOT:PSS solution can be applied to a substrate using a simple casting process such as drop coating, dip coating, inkjet printing, spray coating, and spin coating with low-cost equipment and under ambient conditions. A thin solid layer on a flat surface is made by the drop-casting method wherein the solution is dropped on the surface and allowed to evaporate after a short time. This method is relatively simple and quick; however, it is difficult to generate a homogeneous thin film, and its applicability is limited to small-area thin solid films and coatings because of the limitations of the method. Eslamian et al. [70] fabricated PEDOT:PSS films using the multiple drop-casting method, which have a larger area than those fabricated using the single drop-casting method. They excited the substrate to ultrasonic vibrations to promote droplet spreading and coalescence. They found that the impinging droplets successfully coalesced as time passed, which results in the formation of a thin film. The dip-coating method is another method of applying a PEDOT:PSS solution to a substrate, wherein the substrate is dipped into the PEDOT:PSS solution to deposit the material and then removed by evaporation, which results in the thickness of the coating. The advantages of dip coating are the low cost and ease in adjusting coating thickness. The disadvantages of dip coating are the slowness of the process and the possibility of clogging the screen, which affects the final product [71]. Almarri fabricated a highly conductive and transparent thin film from a composite of PEDOT:PSS and black carbon using the dip-coating method. The transmission modulation of the resulting film was 91% at 550 nm with a coloration efficiency of 45 cm^2^ C^−1^ [72]. Another method of applying PEDOT:PSS to a substrate is inkjet printing. In this method, the materials are transferred to a substrate in a liquid phase. This is achieved in a controlled manner through a noncontact process in which an external signal is applied to the print head. Preston et al. developed an inkjet-printed transparent PEDOT:PSS thin film and used it as a microelectrode. They found that the electrical impedance of the film decreased from 294 to 98 kΩ and the charge storage capacity increased from 6 to 21 mC cm^2^ when the outer diameter of the electrode was increased from 300 to 550 μm [73]. Spin coating is a fantastic process for producing high-quality multilayer thin films for small applications such as solar cells. Producing uniform layers in electronic devices with PEDOT:PSS is a relatively simple, fast, and repeatable approach to this technique. However, the disadvantage of spin coating is that the throughput is relatively low because of the small size of the substrate, and it cannot be scaled up. Further, the actual material consumption in spin coating is very low as the remainder is spun away and wasted. For research purposes, this is usually not a problem; however, for large scale production, it is clearly wasteful. Sanviti et al. [74] fabricated thin PEDOT:PSS nanostructures on an indium tin oxide substrate by spin coating after adding a drop of tetrahydrofuran to the polymer. They found that the obtained samples exhibited distinct conductive regions on the top surface, whereas the nanoelectronic properties of the bulk remained unchanged. Spray coating is another method of applying PEDPT:PSS to a substrate; it has great potential for large-scale production because it has no substrate size limitations, consumes few polymers, and has the potential to replace the traditional spin coating process. Alamer et al. [75] prepared conductive polyester fabric by spray coating with PEDOT:PSS. They found that the sheet resistance of the conductive fabric reached the minimum value of 12.10 Ω/sq at a PEDOT:PSS concentration of 23.4%. They attributed the improvement in the electrical conductivity to the enhancement in uniformity, thickness, and the formation of PEDOT microcrystals in the multiple coatings, which means better conduction paths.

Ko et al. [76] described a straightforward aqueous-solution-based technique to prepare a thermally processable photothermal nanocomposite of PEDOT:PSS and agarose with light-triggered self-healing and good antibacterial activity. The electrical analyses of the films revealed that the electrical conductivity was three orders of magnitude higher than that of the polymer film because of the presence of a silver core. These properties suggest that Ag along with PEDOT:PSS films can be used in a wide range of applications including electro-optics, smart windows, amperometric sensors, and capacitors [77].

Moreno et al. [78] investigated the effect of Ag-NP size on the electrical conductivity of PEDOT:PSS in Ag PEDOT:PSS films by changing the molar ratio of AgNO_3_ to the reducing agent in the presence of PEDOT:PSS. The Ag NPs were prepared using NaBH_4_. The electrical conductivity was found to increase inversely with the concentration of the reducing agent by three orders of magnitude compared with that of pure PEDOT:PSS. The physical properties of CPs such as their mechanical and electrical stabilities are critical for their application in organic devices, where they may be exposed to a wide range of temperatures. High-resolution characterization of the microstructure is required to study PEDOT:PSS films; therefore, the aim of this study is to follow these microstructural changes at different temperatures and relate them to the changes in the macroscopic physical behavior of PEDOT:PSS films. 

In recent decades, numerous methods have been developed to enhance the conductivity of PEDOT:PSS. PEDOT:PSS can be dispersed in water and various polar organic solvents, which allows for the rapid preparation of high-quality PEDOT:PSS films. Further, PEDOT:PSS offers high thermal stability and is highly transparent in the visible region. Dispersion in organic compounds such as high-boiling-point polar organic solvents, ionic liquids, and surfactants, as well as the post-treatment of PEDOT:PSS films with organic compounds such as salts, zwitterions, co-solvents, organic, and inorganic acids, can significantly improve the conductivity of PEDOT:PSS prepared in aqueous solutions [79]. 

Physical processes such as heat and light treatments have been demonstrated to increase the conductivity of PEDOT:PSS, and organic solvents have been found to be crucial elements in vapor drying processes with polar solvents; they provide conductivities greater than 1000 S/cm. Polar solvents affect the conductivity and resistivity of PEDOT:PSS. In this review, a few of these solvents have been investigated. Sulfuric acid is a strong and corrosive acid that possess safety and environmental problems, especially in the industry. The drastic improvement in the conductivity of PEDOT:PSS using non-toxic chemicals is of practical significance [80]. 

DMSO is commonly used to improve the electrical conductivity of PEDOT:PSS thin films because of its specific advantages such as an improved dipole moment, a high dielectric constant, and a relatively high boiling point [81]. Lim et al. [82] prepared the following four samples using different methods: pristine PEDOT:PSS, PEDOT:PSS doped with 5% DMSO, PEDOT:PSS with an immersion, and PEDOT:PSS with dynamic etching. They discovered that the sheet resistivities of the samples decreased, and the conductivity of PEDOT:PSS produced with the dynamic etching method was 1299 S/cm, which was higher than the conductivity of 1100 S/cm of PEDOT:PSS produced using the immersion method. Yildirim et al. [83] applied a combination of experimental and multiscale molecular modeling methods to demonstrate that dimethyl sulfone (DMSO_2_) could be a more environmental-friendly alternative to the commonly used DMSO for the solvent treatment of PEDOT:PSS chains. Moreover, crystalline DMSO_2_ nanowire surfaces left over in the PEDOT:PSS films served as a template for the self-alignment of the PEDOT chains, which further improved the electrical conductivity. DMSO, ethylene glycol, DMSO_2_, and other acidic dopants have been used in the doping and post-treatment of PEDOT:PSS because they appear to be a viable environmental-friendly alternative to conventional additives; DMSO_2_, as a dopant for PEDOT:PSS, can increase the electrical conductivity from 0.2 to 1080 S/cm, which can be attributed to the alignment of the polymer by the crystallization of DMSO_2_ [84]. 

Okuzaki et al. [85] prepared highly conductive microfibers of PEDOT:PSS by wet-spinning, followed by immersion treatment in ethylene glycol. The electrical conductivity of these PEDOT:PSS microfibers was found to drastically improve from 74 to 467 S/cm within 3 min by an immersion treatment. Wet spinning the aqueous PEDOT:PSS dispersion and subsequent immersion treatment in ethylene glycol produced highly conductive microfibers with a diameter of 5 µm. Furthermore, PEDOT/magnetite NPs were used to prepare electrically conductive fabrics with superparamagnetic properties [86]. PEDOT:PSS films dissolve readily when immersed in water, and these effects are exacerbated by vigorous washing. However, the fabric treated with PEDOT:PSS by immersion retained minimal conductivity after ten wash cycles, which indicates the presence of a dyeing effect. The surface resistivities of only 1.7 and 6.4 Ω/square were obtained for dipping and coating, respectively, with dipping yielding more consistent and better conductivity than coating [87].

Wang et al. [88] successfully demonstrated the overall improvement in yarn formation by the introduction of a solvent treatment to the polyvinyl acrylate/PEDOT:PSS solution and the reduction in the electric field strength required for yarn formation, from >2.43 to 1.875 kV/cm, by the addition of solvents such as ethylene glycol and DMSO. In another study, five organic solvents with different boiling points and dielectric constants: Toluene, isopropanol, methanol, dimethylformamide, and DMSO were used in a systematic and fundamental study on the effects of different organic solvents on the PEDOT:PSS structure, and electrical conductivity when used as additives in the initial solution or as post-treatment media [89].

Ahmad et al. [90] prepared PEDOT:PSS films using a sodium dodecylbenzenesulfonate surfactant and conductive ionic liquid and reported high surface electrical conductivity, interlayer spacing, and polymer coating. A small amount of dilute sulfuric acid was added to the PEDOT:PSS solution to produce thin films, which results in a conductivity of approximately 100 S/cm, which is 1400 times higher than those of the original PEDOT:PSS films. A significant increase in the conductivity was not observed when hydrochloric acid, another strong acid, was used for comparison [91,92]. 

Wilson et al. [93] investigated the conductivity of PEDOT:PSS films prepared by inkjet printing or spin coating as a function of the addition of DMSO as a conductivity enhancer and Surfynol as a surfactant. Compared with the effect of a co-solvent, Surfynol, was found to exhibit a negligible effect on the film resistance as a surfactant. Similarly, different surfactants such as Triton-X100, FS-300, and sodium dodecyl sulfate were also added to the ethylene glycol-optimized PEDOT:PSS solution, and extremely low sheet resistance values were obtained with 0.25 wt% FS addition. This suggests that these processes can be very useful in improving the properties of electronic devices [94]. 

Huang et al. [95] measured the conductivity of spin-coated PEDOT:PSS films with heat treatment processes at temperatures ranging from 100–250 °C and different treatment times at 200 °C in both air and N_2_ atmospheres and noted a significant increase in the conductivity after heat treatment, consistent with the surface roughness. In another study, PEDOT:PSS and toluene solutions were studied, which considers their stability, wettability, transparency, and electrochemical properties, and the polarity changes resulting from the dopants using methanol, ethanol, and isopropanol with dipole moments of 1.70, 1.69, and 1.66 D and dielectric constants of 33.0, 24.5, and 18.0, respectively. All PEDOT:PSS solutions in water and toluene demonstrated good wetting properties with angles below 30° for all tested surfaces [96].

Niu et al. [97] applied solvent post-treatments, which includes toluene vapor annealing and ethylene glycol washing to modify the electrical conductivity and work function. Further, surface composition and morphology of PEDOT:PSS thin films were observed. The results indicated that annealing PEDOT:PSS films in a nonpolar toluene vapor environment led to slightly improved electrical conductivities and increased work functions while maintaining the surface composition and morphology.

Ethanol is a safe and easily accessible solvent and the vapor polarity of the solvent is used to explain the process of conductivity enhancement by modifying the surface of the PEDOT:PSS film coated with ethanol, which can reduce the sheet resistance from 130 to 97 Ω/sq. PEDOT:PSS films treated with polar vapors with a low sheet resistance and good optical transmittance have been used as anodes in organic light-emitting devices [98]. Compared with other CPs, PEDOT:PSS possesses advantages in terms of processability, mechanical flexibility, and optical properties, and it is well dispersible in water and polar organic solvents. Several approaches have been explored to enhance the thermoelectric performance of conductive PEDOT:PSS films, which include the addition of a polar solvent and acids and other post-treatments. This paper describes a technique for treating PEDOT:PSS films with a superacid, trifluoromethanesulfonic acid, in methanol to enhance their thermoelectric performance. This treatment was found to result in a sharp increase in the electrical conductivity from 0.7 to 2980 S/cm, and a slight increase in the Seebeck coefficient from 17.6–21.9 µV K^−1^, which results in a power factor of 142 µW m^−1^ K^2^ [99].

## 4. Textiles

E-textiles, which are also known as electronic or smart textiles, are fabrics made from electrically conductive threads that allow the combination of textiles and electronic devices. Wearable sensors, portable storage devices, antennas, and flexible photovoltaic devices have benefited from the introduction of e-textiles as a new technology that can add value to current wearable applications [100]. E-textiles are expected to have a significant impact on sports, military, and aerospace industries because they offer enhanced benefits such as environmental protection, thermal regulation, and energy conduction [101]. Conductive fibers are a crucial component of e-textiles not only because they can be used as lightweight wiring for the basic textile-based electronic components, but also because they can be used to manufacture electronic devices directly on textile fibers [102]. In recent years, the textile industry has focused on improving the functional properties of textiles [103,104].

Fibers or fiber composites with the ability to recognize, operate, rely, communicate, heal themselves, serve themselves, and remember, learn, and perform other functions are called intelligent integrated microelectronic systems. However, most textiles made from dielectric fibers lack these functions. There exist three primary approaches for transferring electronic functions to wearable devices. First, the structural homogeneity of the entire electronic textile system is changed; second, surface coating or printing through modification at the level of fibers and textiles is adopted; and third, microelectronic devices are incorporated into wearable products or into their components such as fibers, threads, or fabrics, which provide the advantage of combining the strengths of several industries, such as textiles, clothing, footwear, jewelry, and electronics [105]. Multifunctional textiles, which possess flame retardant, self-cleaning, antibacterial properties, and ultraviolet blocking properties, and they help improve the quality of life. Electrically conductive textiles with the inherent properties of electromagnetic interference shielding and electrothermal heating are at the forefront among these functional textiles because of their widespread use in biomedical sensors and radiation protective clothing [106].

The evolution of the fibers that transform cloth into electrical devices is crucial for the development of textile electronics. An electronic fiber must undergo a series of tests including material selection, device architecture, wear properties, mechanical stress, environmental impact, and end-user management to be selected as a multifunctional textile [107]. Table 1 provides a comparison between the following assembly methods for manufacturing fibers: dip-coating, spray-coating, drop-coating, and spin-coating methods.

Durán et al. [114] demonstrated the use of biologically produced Ag NPs and their incorporation into materials. For example, cotton textiles impregnated with Ag NPs were found to exhibit antibacterial properties against *Staphylococcus aureus* and *Chromobacterium violaceum* and effectively purify wastewater resulting from the washing process of cotton fabrics. This treatment was based on biosorption and proved to be reasonably effective in eliminating the Ag NPs left behind in the wash water, which results in reduced environmental pollution. Conventional textile finishing processes that endow textiles with different properties do not always produce durable results, and their functionalities are lost after washing or wearing. NPs possess two important properties: (1) large surface area and (2) high surface energy, and they can provide treated textiles with a longer shelf life than conventional materials. These two properties provide greater fabric affinity and increase the longevity of textiles [115].

Stoppa and Chiolerio et al. [116] examined the current advances in the field of smart textiles while focusing on materials and production processes. Each approach has its own advantages and disadvantages, and the aim was to demonstrate a possible trade-off between the flexibility, ergonomics, low energy consumption, integration, and autonomy.

Grancarić et al. [117] focused on CPs, their conductivity mechanisms, and various methods to fabricate electrically conductive textiles for smart textile applications. Various laboratory-scale devices and commercial products incorporating smart textiles based on CPs have been investigated.

Castano and Flatau et al. [118] discussed smart textile transducer elements, textile platforms, application approaches, and fabrication methods. The lightweight and compliant properties of smart textiles have inspired a variety of applications, which includes industrial uniforms, aerospace spacesuit linings, military equipment such as aerospace spacesuits, military equipment for soldiers, and medical patient garments. Various solvents such as shape memory polyurethane, polyester, polyhydroxyproline, polysilane, and various reactive hydrogels such as poly(N-isopropylacrylamide) hydrogels and polythiophene gel have been used to fabricate smart textiles [119].

Guo et al. [120] used PEDOT:PSS wires printed on textiles for wearable electronics; these wires were found to provide a wide range of resistances from tens of kΩ/sq to less than 2 Ω/sq; this allowed tailoring the resistance according to a specific application. The sheet resistance was as low as 1.6 Ω/sq; the breakdown current was 0.37 A. 

### 4.1. Cotton Fabrics

Cellulose is the most widely distributed natural biopolymer and organic polymer on the planet. It is the primary component of cotton fibers [121] and accounts for approximately 1.5 × 10^12^ tons of the total annual biomass production. It is considered an infinite supply of raw materials to meet the increasing demand for eco-friendly and biocompatible goods. Cellulose derivatives are useful in a variety of applications because they can be dissolved in common organic solvents or melted at low temperatures [122]. Cotton fabrics, which are bio-based textiles, have the largest market demand because of their remarkable advantages including softness, comfort, and hydrophilicity [123]. Cotton demonstrates a low resistance owing to the presence of free hydroxyl groups. Therefore, the free hydroxyl groups in cotton are either eliminated or crosslinked with formaldehyde and non-formaldehyde-based crosslinking agents to improve the crease recovery properties [124]. 

According to a study by El-Rafie et al. [125], cotton fabrics were found to exhibit excellent antibacterial activities. Cotton fabrics were coated with a low concentration of Ag NPs to demonstrate this and washed using an eco-friendly approach to determine the amount of silver leached into the washing solution. The Ag NPs were found to endow the cotton fabric with excellent antibacterial properties as well as excellent wash resistance. After 20 washes, the cotton textiles coated with nano-silver exhibited good antibacterial activity.

Rajendran et al. [126] demonstrated that cotton fabrics with excellent antibacterial activities could be produced as shown by the antimicrobial evaluation of the treated fabrics and wash resistance analysis. Treated fabrics were found to exhibit easy care quality and a few of the imparted functional properties such as softness, water, oil repellency, antimicrobial activity, ultraviolet protection, and self-cleaning. Even after ten washes, the effectiveness of the mediated properties remained unchanged [127].

Dong and Hinestroza et al. [128] described two methods for depositing metal NPs on the surface of natural cellulose fibers. In the first method, metal NPs coated with negative citrate ions were prepared and electrostatically attached to cationic cellulose surfaces. In the second method, negative metal complex ions were adsorbed onto cationic cellulose substrates and subsequently reduced. Both methods resulted in a significant surface coverage of the cellulose substrates with metal NPs. 

Karimi et al. [129] reported an innovative approach to achieve multifunctional properties such as self-cleaning, electrical conductivity, ultraviolet blocking, and antimicrobial properties by using graphene oxide and TiO_2_ nanocomposites deposited on the surface of cotton fabrics. They discovered that this approach improved the electrical conductivity and resulted in excellent photocatalytic self-cleaning activities and they could be used for the multifunctional treatment of cotton fabrics by the dip-dry technique. A sol−gel coating of TiO_2_ and surface hydrophobization were used to create bifunctional cotton fabrics with superhydrophobic and ultraviolet-repellent properties. Such fabrics are in a high demand in technical, industrial, medical, military, and daily life applications such as sunshades, shades, bearings, and clothing [130]. Radiofrequency plasma, microwave plasma, or ultraviolet irradiation have been used to activate cotton fibers and introduce negatively charged functional groups to attach TiO_2_ NPs to the textile surface [131]. TiO_2_ NPs were applied to the cotton using a dipping method, wherein the particles were drawn into the inter-fiber spaces by the expansion of the cotton matrix during the initial soaking process [132].

Alamer et al. [133] demonstrated that conductive cotton fabric with a high concentration of PEDOT:PSS can act as a wire in a normal electrical electrode, which can lead to significant advances in the use of conductive fabrics as substitutes for copper. 

DMSO, which affects the conductivity of PEDOT:PSS films, was used in a drop-casting process to fabricate conductive fabrics. This is a viable option because it is straightforward, inexpensive, and safe. The PEDOT:PSS cotton fabric was investigated at temperatures between 30 and 100 °C.

### 4.2. Nanoparticles-Doped PEDOT:PSS Fabric

We studied the effects of nanoparticles, especially silver, gold, and titanium nanoparticles, on the physical properties of cotton fabrics in general and of cotton fabrics coated with conductive materials, which focus on PEDOT:PSS. The composite of nanomaterials and PEDOT:PSS-based smart textiles is now being integrated into textiles to perform a variety of functions, which includes energy generation and storage and stress sensors. Electromagnetic radiation shielding is one of the most important applications of smart textiles by treating the fabric with UV-blocking made with the composite of nanomaterials and PEDOT:PSS in which the TiO_2_ can scatter or absorb UV radiation. Furthermore, this composite in the textile can be used for antibacterial activities and that are beneficial for human health. Das et al. [134] developed a smart electronic textile to shield electromagnetic radiation. They first synthesized gallium nanoparticles embedded in reduced graphene oxide, and they used them as conductive inclusions for PEDOT:PSS and coated them on cotton fabric. They found that the nanocomposite electromagnetic radiation efficiency was reduced by about 34 dB in the frequency range of 8.2 to 12.4 GHz, and the thermal conductivity was reduced by about 44% compared to the original cotton fabric. In another study, Ganguly et al. [135] fabricated a thin coating of graphene nanofibers and iron oxide nanoparticles on nonpolar plastic by photopolymerization. They found that the surface conductivity of the coated films was about 20 S cm^−1^ and the shielding efficiency was about 24 dB in the frequency range of 8.2 to 12.4 GHz. Darvishzadeh and Nasouri [136] deposited nickel on the surface of the carbon fabric. The nickel electroplating process was carried out by the BoxBehnken method. They found that the electroplating process was affected by the applied current density and time, but not by temperature. These two factors affected the electrical conductivity of the conductive fabric. They found that the electrical conductivity, weight gain, and current density at 25 °C and a time of 20 min were 585 S cm^−1^, 38 wt%, and 2.5 A/dm^2^, respectively. In addition, the shielding efficiency of the nickel-coated carbon fabric reached a value of −63.71 dB.

Ag NPs deposited on textiles are of particular interest because of their broad spectrum of antimicrobial activity. In addition, Ag NPs could be used in various applications such as drug delivery strategies, medical device coatings, and diagnostic platforms. Tang et al. [137] modified cotton fabrics with Ag NPs to impart antibacterial activity to the cotton using the sol–gel method, in which the sol–gel contains dodecanethiol-capped silver nanoparticles. They found that the treated cotton exhibited excellent antibacterial activity against *Escherichia coli*, which was because of the reaction of Ag NPs with proteins. In another study, the AgNPs were imparted to the cotton fabric by adjusting pH value using the in situ method. The treated cotton fabric exhibited bright colors and strong antibacterial properties. Perera et al. [138] compared different methods to apply Ag NPs to cotton fabrics. They used two chemical approaches: the ex situ and in situ approaches, and they investigated the effects of the treatment on the physical properties of the cotton fabrics. In both approaches, Ag NPs were prepared from AgNO_3_. They found that Ag-NPs were agglomerated and uniformly distributed on the fabric structure in the ex situ and in situ methods, respectively. Moreover, the samples prepared by both approaches showed antimicrobial activity against *S. aureus* than against *E. coli*, with a higher concentration of AgNO_3_ increasing the antimicrobial activity. The durability of cotton produced by the ex situ method was worse than that of cotton produced by the in situ method after repeated washing. The thickness of the fabrics treated with Ag NPs was the same as that of the untreated fabrics because of the insignificant size of the nanoparticles; however, the weight of the fabrics treated with Ag NPs was significantly higher compared to the untreated fabrics. The untreated and treated fabrics had the same tensile properties. In another study [139], conductive cotton fabrics were prepared using polyaniline derivatives and Ag NPs. First, a plasma activation technique was used to prepare a thin layer of the polyaniline derivative on the cotton surface, and then, Ag NPs were incorporated on the surface. The result showed that the addition of AgNPs to the PANI matrix resulted in an increase in electrical conductivity from 1.2 × 10^−2^ S cm^−1^ to 0.5948 S cm^−1^, which was because of the synergistic effect of AgNPs. In addition, the conductive cotton exhibited antibacterial activity because of the antimicrobial effect of AgNPs against various bacteria. In another study, AgNPs were applied to cotton fabrics using the in situ method, and the diffusion of the nanoparticles on the cotton fabrics was enhanced using carboxymethyl chitosan. The result showed that the cotton fabrics treated with AgNPs exhibited antibacterial behavior and long washing durability against *S. aureus* and *E. coli* even after 20 washing cycles. In addition, the treated cotton showed UV protection compared to untreated cotton [140].

Sensory effects of plasma-activated cotton immobilized with PANi/Ag NPs were observed in the coloration measurements, which led to the introduction of smart sensory fabric. Siavashani et al. [141] fabricated a stretchable cotton/Lycra fabric from nanocomposites of polypyrene, Ag NPs, and PEDOT:PSS. They first coated the nanocomposites onto the fabric by in situ polymerization and then covered it with a PEDOT:PSS solution by dip coating. They found that the electrical conductivity of the fabric increased from 12 to 43 S m^−1^. They attributed the increase in conductivity to the increase in the concentration of pyrrole and silver nitrate. In addition, PEDOT:PSS filled the uncovered portion of the nanocomposites, which results in a continuous coating of the surface. Wang et al. [142] used mist polymerization to prepare a conductive cotton fabric with PEDOT:PSS and Ag NPs. They immobilized Ag NPs on cotton fabric with L-cysteine as a binder and then coated them with PEDOT:PSS. They found that the treated cotton fabric had a sheet resistance of 8.7 Ω/sq and electromagnetic shielding of 27.1 dB. 

Among nanoparticles, AuNPs have received considerable attention in textile research because of their promising physical properties, especially their optical, electronic, and magnetic properties. In addition, AuNPs are used in textiles for electronic and medical applications. Tang et al. [143] modified cotton fabrics with AuNPs using a heating method in which the cotton fabrics were immersed in aqueous HAuCl_4_ solutions with different concentrations to obtain the nanoparticles. They found that the intensity of localized surface plasmon resonance of the treated cotton was proportional to the concentration of AuNPs. Further, they found that the color of the treated fabric did not change upon washing and rubbing. In addition, they tested the antibacterial activity of treated cotton against the Gram-negative bacterium *E. coli* and found that the sample exhibited excellent antibacterial activity. In addition, the AuNPs improved the UV-blocking ability of the cotton fabric. In another study, AuNPs were synthesized from chloroauric acid using a greener method, and then applied to cotton fabric using the pad-dry-cure method [144]. 

Mattana et al. [145] prepared conductive cotton fibers with a combination of PEDOT and Au NPs. They first applied Au NPs to the cotton surface and then soaked the cotton fiber in a PEDOT solution to form a thin layer. They showed that the electrical conductivity and mechanical properties of the PEDOT/cotton fiber were improved with Au NPs compared with the PEDOT/cotton fiber or Au NPs/cotton fiber. Moreover, the Au NPs/PEDOT cotton fibers were used to fabricate organic electrochemical transistors and organic field effect transistors. Xin et al. [146] fabricated a flexible photo-thermoelectric generator with hybrid PEDOT and Au NPs; they found that the photo-thermoelectric generator has an output voltage of 34 mV and a maximum output power of 146 nW under solar irradiation, which is larger than the output voltage and output power of the photo-thermoelectric generator fabricated without Au NPs. 

TiO_2_ NPs are increasingly used in a variety of applications such as dye toxicity reduction, wastewater treatment, and aerospace. In addition, the electronic textile industry is another important application for the use of TiO_2_ NPs because of their high photocatalytic performance under UV light, non-toxicity, cheapness, and chemical stability [147]. Perelshtein et al. [148] studied the effect of UV light irradiation on TiO_2_ NPs deposited on cotton fabrics; they found that UV irradiation promoted the stability of TiO_2_ NPs, imparted antimicrobial properties to them, and exhibited significant antimicrobial activity against microorganisms. Abidi et al. [149] used a sol–gel method to prepare cotton fabrics with TiO_2_ NPs. They found that the UV scattering of the cotton fabric was enhanced, and they found that increasing the concentration of TiO_2_ NPs resulted in increased UV protection. Further, they showed that the treated cotton fabrics had excellent durability, which indicates good adhesion between the coating and fabric surface. Karimi et al. [150] fabricated cotton fabric with a nanocomposite of graphene oxide and TiO_2_ NP by first creating a thin graphene oxide film on the cotton fabric and then immersing it in an aqueous titanium trichloride solution. They found that the treated cotton fabric exhibited significant photoefficiency under UV and sunlight irradiation. In addition, they found that the nanocomposite-treated cotton fabric exhibited low toxicity and antibacterial activity. In another study, cotton fabric was prepared with platinum and TiO_2_ NPs by a simple dip-coating method. The treated cotton fabric exhibited self-cleaning properties and higher photocatalytic activity for methyl orange and stain degradation [151]. Noman et al. [152] applied green synthesized TiO_2_ NPs to cotton fabrics and explained the stability of TiO_2_ NPs using UV light. They found that the self-cleaning efficiency of the treated cotton was proportional to the amount of TiO_2_ NPs. Further, they also showed that the stability of TiO_2_ NPs on the cotton surface was affected by the UV light, which caused the covalent bonding. In a recent study [153], the mixture of TiO_2_/Ag NPs deposited on cotton fabrics was investigated and compared with the individual deposition of TiO_2_ NPs and Ag NPs on cotton. The results showed that cotton fabrics treated with Ag/TiO2NPs exhibited higher values in both antimicrobial activity and UV protection, and in improvements in tensile strength and elongation properties compared to individual deposition.

The mixture of PEDOT:PSS and TiO_2_ NPs was coated on a glass substrate and used as a dye-sensitized solar cell counter electrode. The effect of the diameter of TiO_2_ NPs mixed with PEDOT:PSS on the performance of the electrodes was investigated. The result showed that high efficiency of 8.49% was obtained when the diameter of TiO_2_ NPs was large, which was attributed to the improvement of the active area of PEDOT:PSS and the reduction of the charge transfer resistance of the film [154]. Ramesh et al. [155] fabricated nanofibers from a hybrid nanocomposite of PEDOT:PSS and TiO_2_ NPs using peroxotitanic acid, which serves as both an oxidant and a source of TiO_2_. They observed that the PEDOT:PSS/TiO_2_ NPs nanofibers were stable up to 315 °C. Further, they fabricated a supercapacitor with a specific capacitance of 162 F/g at a current density of 0.25 A/g using these nanofibers. Cesarini et al. [156] fabricated sensors from a hybrid of PEDOT:PSS and TiO_2_ NPs using inkjet printing technology; they first printed five layers of TiO_2_ NPs ink on the polyethylene naphthalate substrate, and then, after curing, they printed the PEDOT:PSS layer. They exposed the sample to UV light and found that the electrical conductivity was high in the dark, which was caused by the recombination between the electrons photogenerated by the TiO_2_ NPs and the holes in PEDOT:PSS. In another interesting study, Berendjchi et al. [157] fabricated dye-sensitized solar cells on a flexible textile substrate using PEDOT:PSS, graphene oxide, and TiO_2_ NPs. The working and counter electrodes of the solar cells were fabricated by coating graphene oxide and PEDOT:PSS in PET and PET /polyamide fabrics, and TiO_2_ NPs were used as a sintered layer. They found that the photoelectric characteristic of the PEDOT:PSS/graphene oxide/TiO_2_ NPs solar cell fabric was lower than that of the standard cell with high flexibility.

### 4.3. Toxicity of Metal Nanoparticles in Terms of Fabrics

Ag NPs and TiO_2_ NPs are the most commonly used nanomaterials in clothing. Ag NPs are embedded in clothing to give it antibacterial properties and prevent bacteria from causing clothing odor attributed to sweating. In addition, TiO_2_ NPs incorporated into garments could provide UV protection for clothing, similar to sunscreens. However, using these nanoparticles to provide fresh-smelling clothing and UV protection may not be entirely safe. Scientists are concerned that these particles, which come in different shapes and sizes, may behave differently in the biological systems of our bodies. It is more likely that these nanoparticles are transported through the skin or cause toxicity in our body. Despite the increasing commercialization of Ag NPs, little is known about their effects on the environment. Further, the fabrics may lose some of the nanoparticles after washing. Pasricha et al. [158] studied the amount of Ag NPs released from the textiles into the water during washing. They found that the number of nanoparticles released in the wash water depended on the type of textiles used. They treated the wash water with bacteria to eliminate the Ag NPs to prevent damage to the environment.

## 5. Conclusions

PEDOT:PSS, the most successful commercially available polymer, demonstrates outstanding properties such as tunable electrical, optical, electronic, and photoelectronic properties, as well as a relatively high electrical conductivity, light weight, easy fabrication, and surface modification. In addition, it is well known for its good water dispersibility, controllable miscibility, excellent mechanical flexibility, satisfactory stretchability, high optical transparency, high and stable electrical conductivity, versatile film-formation techniques, and low cost. These properties render it a promising organic polymer for a wide range of academic and industrial applications.

In this study, the effects of metallic Ag NPs, Au NPs, and Ti NPs on the physical properties of PEDOT:PSS were investigated. The properties of PEDOT:PSS were found to strongly depend on the synthesis technique, doping, post-treatment, and the composite material. It is expected that PEDOT:PSS will continue to be an important electronic material in current technology development, as it has been for several years. Electronic textiles or smart textiles are composed of electrically conductive threads that allow textiles and electronic devices to be interconnected. Cotton fabrics, which are bio-based textiles, have the largest market demand because of their numerous advantages such as softness, comfort, and durability. In addition, cotton possesses a low resistance because of its free hydroxyl groups.

## Figures and Tables

**Table 1:** Comparison of assembly methods for manufacturing fibers: dip-coating, spray-coating, drop-coating, and spin-coating methods.

Methods	Advantages	Disadvantages	References
Dip-Coating method	No additional electrical instrument is required Design of conductive layers on the nanoscale It is a straightforward, cost-effective, and energy-efficient solution	Compatibility in the production of long fibers is low The speed of manufacture is slow The film may or may not solidify during the dip coating process	[107,108]
Spray-Coating method	Cost-effective, adjustable thickness, and large area coverage It can produce a very thin, dense, and stable film on the surface of the substrate	Compatibility in the production of long fibers is low The conductive layer is not uniform	[107,109]
Drop-Coating method	A simple procedure, no material waste is produced, and no requirement for special equipment It is commercially available	Limitations in large area coverage and thickness control, and poor uniformity	[110,111]
Spin-Coating method	The most commonly used method due to the ease of use and production of low-material waste No special equipment is required	The inefficiency of the material used The method uses only approximately 2 to 5 of the materials spread on the substrate The remaining 95 to 98 are discarded	[110,112,113]

## Data Availability

Not applicable.
